# Perspectives of primary care physicians and pharmacists on interprofessional collaboration in Kuwait: A quantitative study

**DOI:** 10.1371/journal.pone.0236114

**Published:** 2020-07-20

**Authors:** Abdullah Albassam, Hamad Almohammed, Malak Alhujaili, Samuel Koshy, Abdelmoneim Awad

**Affiliations:** 1 Department of Pharmacy Practice, Faculty of Pharmacy, Kuwait University, Kuwait, Kuwait; 2 Drug and Food Control Administration, Ministry of Health, Kuwait, Kuwait; 3 Department of Pharmacy, Jaber Alahmad Polyclinic, Ministry of Health, Kuwait, Kuwait; College of Pharmacy & Health Sciences, UNITED STATES

## Abstract

Collaborative practice between physicians and pharmacists has a positive effect on healthcare outcomes. Understanding the local data related to this collaboration is vital in establishing efficient collaboration. Therefore, this study was designed to assess the collaborative relationships between physicians and pharmacists working in the primary healthcare centres regarding their attitudes and experiences, preferred methods of communication, perceptions related to the role of pharmacists, areas of potential further collaboration, and perceived barriers. A cross-sectional study was conducted using two parallel pretested self-administered questionnaires on a sample of 518 randomly selected physicians and pharmacists. Descriptive and comparative analyses were used in data analysis. The overall response rate was 86.3%. Although over 98% of respondents agreed that physician-pharmacist collaboration improves patient outcomes, more than half of the physicians (52.1%) and pharmacists (55.7%) had never practised collaboratively. Both groups preferred to communicate face-to-face (76.7%) or via telephone (76.5%). Both professions showed good agreement on pharmacists’ roles related to managing side effects, improving adherence, assisting in dosage adjustment, providing advice regarding drug interactions, and providing drug information to physicians. They indicated disagreements on the importance of dispensing of prescriptions and providing advice to physicians regarding modification of drug therapy. Both groups expressed overall positive perceptions of the potential for further collaboration in areas related to the clinical roles of pharmacists, which were significantly higher among those with practice experience of < 10 years and those aged < 40 years (p<0.05). The top four perceived barriers to collaborative practice were lack of time (84.1%), lack of financial compensation (76.3%), lack of face-to-face communication (68.9%), and the possible fragmentation of patient care by the involvement of multiple healthcare professionals (68.9%). The present findings provide valuable input that could be a catalyst to enhance or establish physician-pharmacist collaboration in primary healthcare settings in Kuwait.

## Introduction

Collaborative practice is required due to the increased chronic disease burden, the aging population, the expansion of sophisticated therapeutic modalities, and rapidly rising healthcare costs [1]. The provision of safe and effective patient care needs multiple healthcare professionals to collaborate efficiently with clearly defined roles and responsibilities. The World Health Organization defined interprofessional collaborative practice as “*a situation when multiple health workers from different professional backgrounds provide comprehensive services by working with patients*, *their families*, *carers and communities to deliver the highest quality of care across settings*” [2]. There is consensus that interprofessional collaboration interventions can improve healthcare processes and patient outcomes. This is especially important in the collaborative relationship between pharmacists and physicians [1]. Pharmacists have been recognized as crucial members of such collaborative practice models due to the increasing complexity of medication therapies, the cost of medication-related morbidity, and the increasing costs of healthcare, which underscore the need for effective working relationships between pharmacists and physicians to improve patient care [3].

The professional roles and responsibilities of the pharmacist have markedly evolved worldwide in recent decades from the traditional role of compounding and dispensing medications to more patient-centred care. Pharmaceutical care is a practice philosophy in which pharmacists collaborate directly with other healthcare professionals and with the patient to optimize medication use through identifying, resolving and preventing medication-related problems [4]. The practice of clinical pharmacy embraces the philosophy of pharmaceutical care [5]. In Kuwait, more than half of the pharmacists work in the governmental sector including hospitals, primary healthcare centres, secondary care hospitals, tertiary care specialized hospitals and centres, pharmaceutical services administration, drug regulation, medical stores administration, and academia. The implementation of clinical pharmacy practice in the government healthcare system is limited to dispensing and pharmacy administration (e.g., stock management, medication orders, and record keeping) with minimal clinical roles largely in the hospitals. These clinical roles included providing drug information, participating in the development of clinical guidelines/protocols, participating in multidisciplinary patient care rounds, and maintaining pharmaceutical care plans, which rely mainly on individual efforts made by some motivated pharmacists. Advancements in pharmacy practice in the private community pharmacies is slower and lag far behind the improvements to the clinical practice in the government healthcare facilities [6–10].

The integral component for the implementation of this collaborative practice is that physicians and pharmacists must assume shared responsibility for decisions using their own specialties and skills to achieve the best patient outcomes instead of viewing clinical decision-making as competitive [4]. Several studies have demonstrated the benefit of a pharmacist/physician collaborative practice model in improving the management of chronic diseases such as hypertension, dyslipidemia, arrhythmias, heart failure, diabetes, asthma, osteoporosis, and psychiatric disorders. The benefits of collaboration include increased medication appropriateness, decreased occurrence of medication-related problems, medication errors and adverse drug reactions, increased communication with patients, improved patients’ medication knowledge and adherence, decreased morbidity and mortality, improved patient health outcomes, reduced physician workload, and decreased healthcare costs [11–19]. As a result of this mounting evidence, there is a growing call for increased pharmacist-physician collaboration [20].

Despite the emerging evidence of its benefits, the implementation of pharmacist-physician collaboration can be challenging due to substantial barriers, including physicians’ negative attitudes, uncertainty regarding pharmacists’ clinical training and judgement, lack of communication, patients’ negative perceptions, and logistic and financial issues, which were reported by studies from the USA, Canada, United Arab Emirates (UAE), Iran and Slovakia [11,21–27]. Numerous facilitators to establish effective physician-pharmacist collaboration have been recommended, including awareness and trust building, appropriate training of pharmacists, clear professional role definition and guidelines, improving patients’ perceptions about pharmacists, identifying patients who may benefit from pharmacist intervention, pharmacists’ access to patient records, and effective communication [11,22,23].

Several studies have focused on the effects of physician–pharmacist collaboration on patient outcomes, and on the development of conceptual models to describe the stages and characteristics of collaboration and integration between physicians and pharmacists and services. However, less attention has been devoted so far to comparing the attitudes, experience, and perceptions of the collaborative professionals, or to identifying the matching viewpoints between their collaborative preferences or barriers. Evidence from studies performed in the USA, New Zealand, Germany, Slovakia and Iraq shows that key factors for effective physician-pharmacist collaboration are positive attitudes (trustworthiness) on both sides and their agreement in perceptions of usefulness (experience), and role specification (preferences and barriers) toward collaborative practice [27–32]. Hence, understanding these attributes to collaboration between pharmacists and physicians is essential for establishing efficient collaboration that may further optimize the delivery of healthcare services.

Quantitative and qualitative studies have been performed worldwide to investigate the attitudes, experience, preferences and barriers to physician-pharmacist collaboration [22,23,25–27,31,33–38]. However, few such studies have been conducted in the Middle Eastern region: three qualitative studies (Qatar and UAE) [23,34] and two quantitative studies (Iraq and Iran) [26,31]. However, there are no similar published studies to date in Kuwait. Understanding the local data related to physician-pharmacist collaboration, which may be different from that reported in international studies, is vital in establishing efficient collaboration to further optimize the delivery of healthcare services. Therefore, this study was designed to assess the collaborative working relationships between primary care physicians and pharmacists in terms of their attitudes and experiences with collaborative practice, preferred methods of communication in collaborative practice, perceptions related to the professional role of pharmacists, areas of potential further collaboration, and perceived barriers to collaborative practice.

## Materials and methods

### Study area

Kuwait is a Middle-Eastern country with an area of 17,820 km^2^ and an estimated total population of 4.2 million people (2018 estimate) [39]. In Kuwait, the healthcare system comprises a public and a private sector. The public sector is the largest provider of healthcare that provides comprehensive advanced health services free of charge for the Kuwaiti nationals. A public insurance scheme exists to provide the same services for the non-Kuwaitis at reduced cost. The private healthcare facilities have shorter waiting times, but the costs tend to be higher than the public sector. Private sector accepts citizens and non-citizens with private health insurance and those who are uninsured. The public sector consists of primary, secondary, and tertiary levels of healthcare delivery. Primary care is provided through healthcare centres (also named as polyclinics) disseminated over the six governorates of Kuwait. The primary healthcare centres offer health services including medical care from general practitioners, dentistry, maternity care, nursing care, preventive care, family medicine and pharmaceuticals. Also, primary care is responsible for coordinating patient care among specialists at the secondary and tertiary levels of care. Each primary healthcare centre has a designated pharmacy led by full-time working pharmacists. All medications dispensed at these primary care centres must be ordered by a physician. The traditional roles of outpatient prescription dispensing and patient counselling are the main services that pharmacists provide at the primary care centres. The majority of these pharmacists are baccalaureate graduates, while few have advanced degrees mainly master in clinical pharmacy.

Secondary healthcare services are provided by seven general public hospitals, each of which provides a full outpatient service as well as a 24-hour emergency service. Secondary care is where most patients are referred when they have a medical condition that cannot be handled at the primary care level. When patients need a higher level of specialty care within the hospital, they are referred to a tertiary care. Tertiary health care services are provided through public specialized healthcare facilities that focus on specific conditions, and these include maternity hospital, psychiatry hospital, Ibn Sina hospital (neurology, neurosurgery and pediatric surgery), chest diseases hospital, organ transplant centre, cancer control centre, ophthalmology centre, burn centre, and allergy centre. Pharmacists at the secondary and tertiary levels have established different branches to accommodate services near to clinics (outpatient pharmacy), emergency departments (emergency pharmacy), and the central pharmacy to serve inpatients. This was done to reduce waiting times and provide a better opportunity for patient counselling. Hospital pharmacists often possess an advanced degree and tend to have a higher level of practice compared to that of primary care pharmacists and community pharmacists in Kuwait. There are power differentials from the hierarchical structure within the medical professions in the healthcare system in Kuwait, where physicians occupy the higher level in the medical hierarchy.

### Study design and population

A descriptive, cross-sectional survey was conducted among physicians and pharmacists working in the primary healthcare centres in Kuwait. Ethical approval for this study was obtained from the Ministry of Health Ethical Committee, Kuwait.

Physicians and pharmacists employed in the healthcare facilities in Kuwait have diverse educational backgrounds, with education and training from Kuwait, other Middle Eastern countries, as well as other countries such as the United States of America, Canada, United Kingdom, and India. Faculty of Medicine and Faculty of Pharmacy at Kuwait University are the only medical faculties in Kuwait. Medical students receive their formal education over seven years and graduate with the Doctor of Medicine (MD) degree. Students in the faculty of pharmacy complete their formal education over five years and graduate with a Bachelor of Pharmacy (BPharm) degree. The undergraduate pharmacy curriculum is designed to develop students’ professional abilities to make rational and evidence-based clinical decisions. Pharmacy students undertake experiential training (clerkships) supervised by professional pharmacy and medical preceptors in various healthcare settings during the fourth and fifth years of their study. In 2016, the Faculty of Pharmacy started a two-year add-on Doctor of Pharmacy (PharmD) program to provide advanced clinical skills and practice experiences to provide optimal clinical pharmacy services to patients.

The sample size was based on the assumption that the proportion of responses to most of the main questions would be 50%, due to the fact there are no previous similar studies from Kuwait. It was determined using the Raosoft sample size calculator using a margin of error of 5% and a confidence interval of 95%, for a target population size of 561 physicians and 359 pharmacists currently practicing in the primary healthcare centres [40]. The minimum sample size estimated for the study was 229 physicians and 186 pharmacists. Assuming a response rate of 80%, a sample size of 286 physicians and 232 pharmacists was enrolled in the study. They were randomly selected from all primary healthcare centres in all governorates in Kuwait using stratified sampling to determine the proportional numbers representing the study population from each governorate, followed by systematic random sampling using the lists containing all names of licenced physicians and pharmacists working in primary healthcare centres.

Data were collected anonymously via a self-administered survey. The selected physicians and pharmacists were contacted face-to-face and provided with an explanation about the aim of the study. They were free to decline to participate. Those who agreed to participate in the study were given the questionnaires by hand, which were completed anonymously and collected from them within 1–2 weeks. They were assured of confidentiality and gave written consent to contribute to the study.

### Study questionnaires

A literature review was performed to identify published studies related to physicians’ and pharmacists’ collaborative practice. The study surveys were adapted from validated questionnaires that were previously used in Canada for pharmacists and physicians [25]. The content validity of the adapted questionnaires was established by a research group at Kuwait University. Face validity of the surveys was assessed with ten pharmacists and ten physicians to ensure clarity of questions. Both questionnaires were pretested for content, design, readability, and comprehension with five pharmacists and five physicians, and suitable amendments were made accordingly so that the questionnaires were simple to understand and answer, yet gave accurate data.

The pre-tested questionnaires consisted of six sections and contained both open-ended and close-ended questions. For five sections, both physicians and pharmacists were asked the same questions. The first section included three questions to provide information about the demographic characteristics of respondents. Section two consisted of three items to provide information about respondents’ attitudes towards collaborative practice and five items about the respondents’ experience with collaborative practice. The third section included five items to determine the preferred methods of communication for collaborative practice. The fourth section was related to the perceived role of pharmacists working in primary healthcare centres. In this part, pharmacists were asked to indicate their level of agreement with eight statements related to specific pharmacists’ roles, whereas physicians were asked to rank the same list of roles in order of importance. The fifth section included seven items to determine respondents’ perceptions towards areas where more collaboration is needed. The final section included nine items to determine the barriers to collaborative practice. The items of the questionnaires and their quantified responses are presented in tables in the results section.

### Statistical analysis

Data were analysed using the Statistical Package for Social Sciences (IBM SPSS Statistics for Windows, version 23, Armonk, NY: IBM Corp). The responses for most of the questions were measured using a five-point Likert scale (strongly agree, agree, neutral, disagree, and strongly disagree). For ease of interpretation in the text, those who answered “strongly agree” or “agree” were classified as having agreed, and those who answered “strongly disagree” or “disagree” as having disagreed. Normality of distributions was determined using the Shapiro-Wilk and Kolmogorov-Smirnov tests, which revealed that the data were not normally distributed. The study participants’ responses were presented as percentages (95% confidence intervals; CI), means (standard deviation-SD) and medians (Interquartile range-IQR). The internal consistency reliability for the sections to assess the respondents’ attitudes towards collaborative practice and perceptions towards areas for further collaboration was assessed using Cronbach’s α test. Both sections demonstrated adequate internal consistency, with the test results as follows: three items for attitudes (0.81) and seven items for perceptions (0.90). Based upon these measures, the overall attitudes and overall perceptions were reported as mean (SD) and median (IQR). The Mann–Whitney test was used because the data was not normally distributed, to evaluate the differences in the overall scores for attitudes and perceptions between two groups of independent variables (profession: physicians vs. pharmacists; age: < 40 years vs. ≥ 40 years; gender: male vs. female; practice experience: < 10 years vs. ≥ 10 years). Data from the responses to evaluate the respondents’ experience with collaborative practice, preferred methods of communication for collaborative practice, and barriers to collaborative practice were compared between the physicians and pharmacists using chi-square tests. Statistical significance was accepted at a p value < 0.05.

## Results

### Demographic characteristics

A total of 518 physicians and pharmacists were approached to contribute to the study, 447 of whom agreed and completed the questionnaires (a response rate of 86.3%). Of the study participants, 230 (51.5%) were pharmacists and 217 (48.5%) were physicians. The median (IQR) age of respondents was 36 (13) years [mean (SD): 38.2 (9.9) years]. Table 1 presents the respondents’ demographic characteristics.

**Table 1:** Respondents’ demographic characteristics (n = 447).

Variable	Pharmacists n = 230	Physicians n = 217	Total n = 447
	n (%)	n (%)	n (%)
Age (Years)*			
*< 40*	174 (75.7)	103 (47.5)	227 (50.8)
*≥ 40*	56 (24.3)	107 (49.3)	163 (36.5)
Gender*			
*Male*	100 (43.5)	87 (40.1)	187 (41.8)
*Female*	130 (56.5)	126 (58.1)	256 (57.3)
Experience (Years)*			
*< 10*	93 (40.4)	70 (32.3)	163 (36.5)
*≥10*	137 (59.6)	139 (64.1)	276 (61.7)

* Percentage may not total 100% due to some missing responses

### Attitudes and experience with collaborative practice

Both professions expressed high positive overall attitudes towards interprofessional collaboration. Over 95% of the study participants agreed that collaborative practice can result in improved patient outcomes (Table 2). Nevertheless, this was not a routine role in their practice, since 53.9% (n = 241; 95% CI: 49.2–58.6) of both professions (physicians n = 113; 52.1%; pharmacists n = 128; 55.7%) reported that they had never practised collaboratively in the past. While 46.1% (n = 206; 95% CI: 41.4–50.8) of respondents indicated participating during the past in a collaborative practice in a formal arrangement, almost one-quarter (n = 48; 23.3%; 95% CI: 17.8–29.8) of them indicated that they had frequently or always practiced collaboratively. Table 3 shows the frequency of collaboration in the past. Females expressed significantly higher positive overall attitudes towards collaborative practice compared to males (p = 0.008). There were no significant differences in the overall attitudes between physicians and pharmacists (p = 0.72), those aged < 40 years and ≥ 40 years (p = 0.67), and those with practice experience of < 10 years and ≥ 10 years (p = 0.32). Physicians were found to be significantly more likely to report that they had rarely collaborated with pharmacists in the past (p = 0.007).

**Table 2:** Respondents’ attitudes towards collaborative practice (n = 447).

Statement	Response* (%)				Mean (SD)		Median (IQR)	
	Disagreed		Agreed					
	Phar	Phys	Phar	Phys	Phar	Phys	Phar	Phys
Collaboration with other healthcare professionals improves patient outcomes	0.9	0.0	99.1	99.5	4.9 (0.4)	4.8 (0.4)	5.0 (0.0)	5.0 (0.0)
Collaboration between pharmacists and physicians improves patient outcomes	0.9	0.0	98.3	98.6	4.8 (0.5)	4.8 (0.4)	5.0 (0.0)	5.0 (0.0)
I would consider collaborating with pharmacists/physicians to improve patient outcomes	1.7	0.0	94.8	95.9	4.6 (0.7)	4.6 (0.6)	5.0 (1.0)	5.0 (1.0)
Overall attitudes					4.8 (0.5)	4.8 (0.0)	5.0 (0.0)	5.0 (0.0)

* Responses rated on a Likert scale ranging from 1 = “strongly disagree” to 5 = “strongly agree”.

Phar: Pharmacists; Phys: Physicians

**Table 3:** Respondents’ experience with collaborative practice (n = 206).

Statement	Pharmacists (n = 102) n (%)	Physicians (n = 104) n (%)	Total (n = 206) Frequency (%;95% CI)	p-value
I have rarely collaborated with pharmacists/physicians in the past	26 (25.5)	46 (44.2)	72 (35.0; 28.5–41.9)	0.007
I have sometimes collaborated with pharmacists/physicians in the past	46 (45.1)	40 (38.5)	86 (41.7; 35.0-, 48.8)	0.41
I have frequently collaborated with pharmacists/physicians in the past	13 (12.7)	9 (8.7)	22 (10.7; 7.0–15.9)	0.47
I have always collaborated with pharmacists/physicians in the past	17 (16.7)	9 (8.6)	26 (12.6; 8.6–18.1)	0.13

### Preferred methods of communication for collaborative practice

In relation to communication for collaborative practice, over three-quarters of respondents preferred face-to-face or telephone communication over social media or paper or fax correspondence (Table 4). Pharmacists were significantly more likely to prefer face-to-face communication compared to physicians (p = 0.007). The preferences of both groups towards other methods of communications were not significantly different (p > 0.05).

**Table 4:** Preferred methods of communication for collaborative practice (n = 447).

Method	Pharmacists (n = 230) n (%) Agreed	Physicians (n = 217) n (%) Agreed	Total (n = 447) n (%) Agreed	p-value
Face to face	189 (82.2)	154 (71.0)	343 (76.7)	0.007
Phone	178 (77.4)	164 (75.6)	342 (76.5)	0.73
Social Media	152 (66.1)	142 (65.4)	294 (65.8)	0.96
Paper	94 (40.9)	90 (41.5)	184 (41.2)	0.97
Fax	38 (16.5)	33 (15.2)	71 (15.9)	0.80

### Perceptions of the professional role of pharmacists

Pharmacists were asked to indicate their level of agreement to eight tasks that were components of their role to improve patient care (Table 5). There was a high extent of agreement (> 90%) among pharmacists regarding four tasks, namely dispensing prescriptions (96.1%), patient counselling about their prescriptions (95.7%), helping to improve patient adherence (93.9%), and helping to manage medication side effects of drug therapy (92.6%). Providing advice to physicians regarding modification of a patient’s drug therapy was ranked with the lowest level of agreement (20.0%).

**Table 5:** Pharmacists’ perceptions of the professional role of the pharmacist *(in descending order according to percentage agreeing)* (n = 230).

Professional Role of Pharmacist	Agreed n (%; 95% CI)
Dispensing prescriptions	221 (96.1; 92.5–98.1)
Patient counselling about their prescriptions	220 (95.7; 91.9–97.8)
Helping to improve patient adherence	216 (93.9; 89.8–96.5)
Helping to manage side effects of drug therapy	213 (92.6; 88.2–95.5)
Assisting in medication dosage adjustment	171 (74.4; 68.1–79.7)
Providing advice regarding drug interactions	164 (71.3; 64.9–77.0)
Providing drug information to physicians to assist in decision-making regarding a specific patient’s drug therapy	145 (63.0; 56.4–69.2)
Providing advice to physicians regarding modification of a patient’s drug therapy	46 (20.0; 15.2–25.9)

Physicians were asked to rank the same eight pharmacists’ tasks to improve patient care. Table 6 shows the physicians’ perceptions of the importance of the same pharmacists’ professional roles. The most frequently considered by the physicians to be among the top three most important pharmacist roles were helping to manage side effects of drug therapy (45.6%), assisting in medication dosage adjustment (39.6%), helping to improve patient adherence (38.3%), providing advice regarding drug interactions (36.4%), and patient counselling (31.8%). Dispensing prescriptions was ranked as the least important role (16.1%).

**Table 6:** Physicians’ perceptions of the importance of the professional role of pharmacists *(in descending order according to frequency at which each was considered to be among the top three most important pharmacist roles)* (n = 217).

Professional Role of Pharmacist	Most important n (%; 95% CI)
Helping to manage side effects of drug therapy	99 (45.6; 38.9–52.5)
Assisting in medication dosage adjustment	86 (39.6; 33.1–46.5)
Helping to improve patient adherence	83 (38.3; 31.8–45.1)
Providing advice regarding drug interactions	79 (36.4; 30.1–43.2)
Patient counselling about their prescriptions	69 (31.8; 25.8–38.50)
Providing advice to physicians regarding modification of a patient’s drug therapy	56 (25.8; 20.2–32.3)
Providing drug information to physicians to assist in decision-making regarding a specific patient’s drug therapy	53 (24.4; 19.0–30.8)
Dispensing prescriptions	35 (16.1; 11.6–21.9)

### Areas of further collaboration between pharmacists and physicians

Respondents were asked about their perceptions towards potential further collaboration in certain areas to provide patient care. Over four-fifths of respondents agreed that there was potential for more collaboration in all areas identified (Table 7). However, there were significant differences in the level of agreement for two of these areas, namely helping to improve patient adherence and assisting in medication dosage adjustment. The proportions of physicians who agreed that there was potential for further collaboration in these two areas were significantly higher compared to pharmacists (p<0.05). In general, physicians indicated that they would like to collaborate more in the areas of helping to improve patient adherence, and assisting in medication dosage and adjustment, followed by patient counselling, providing drug information to help select a medication, helping in the management of side effects of drug therapy, and making recommendations to modify a patient’s drug therapy. This is in contrast to pharmacists, who indicated that they would like to collaborate more in patient counselling and helping in the management of side effects of drug therapy, followed by providing advice regarding drug interactions, helping to improve patient adherence, providing drug information to select a medication, assisting in medication dosage adjustment, and making recommendations to modify a patient’s drug therapy.

**Table 7:** Respondents’ perceptions of areas of further collaboration between pharmacists and physicians (n = 447).

Area of further collaboration	Agreed n (%)		P-Value	Mean (SD)		Median (IQR)	
	Phar	Phys		Phar	Phys	Phar	Phys
Patient counselling	223 (97.0)	202 (93.1)	0.09	4.6 (0.6)	4.4 (0.6)	5.0 (1.0)	5.0 (1.0)
Helping in the management of side effects of drug therapy	221 (96.1)	201 (92.6)	0.17	4.6 (0.6)	4.4 (0.7)	5.0 (1.0)	4.0 (1.0)
Making recommendations to modify a patient’s drug therapy	197 (85.7)	196 (90.3)	0.17	4.3 (0.8)	4.3 (0.7)	5.0 (1.0)	4.0 (1.0)
Assisting in medication dosage adjustment	201 (87.4)	210 (96.8)	<0.001	4.4 (0.8)	4.5 (0.6)	5.0 (1.0)	4.0 (1.0)
Providing drug information to help select a medication	202 (87.8)	202 (93.1)	0.08	4.4 (0.7)	4.4 (0.6	5.0 (1.0)	4.0 (1.0)
Providing advice regarding drug interactions	207 (90.0)	200 (92.2)	0.53	4.4 (0.7)	4.4 (0.7)	5.0 (1.0)	4.0 (1.0)
Helping to improve patient adherence	203 (88.3)	214 (98.6)	<0.001	4.4 (0.7)	4.6 (0.5)	5.0 (1.0)	5.0 (1.0)
Overall perceptions				4.4 (0.7)	4.4 (0.6)	5.0 (1.0)	4.0 (1.0)

* Responses rated on a Likert scale ranging from 1 = strongly disagree to 5 = strongly agree.

Phar: Pharmacists; Phys: Physicians

Higher overall positive perceptions were found among pharmacists compared to physicians (p = 0.001) and in those aged < 40 years compared to those ≥ 40 years (p = 0.007). There were no significant differences in overall perceptions of the potential for more collaboration between males and females (p = 0.65) or between those with practice experience of <10 years and ≥10 years (p = 0.27).

### Barriers to collaborative practice

Table 8 presents the level of agreement of both groups regarding the barriers to collaborative practice. Over two-thirds of the study participants agreed that the top four most significant barriers were lack of time (84.1%), lack of financial compensation (76.3%), lack of face-to-face communication (68.9%), and possible fragmentation of patient care by the involvement of multiple healthcare professionals (68.9%). There were significant differences between the responses of the two groups in relation to four barriers, where the proportion of pharmacists who agreed on these barriers was significantly higher than that of physicians (p<0.05). These four barriers were the lack of face-to-face communication, possible fragmentation of patient care through the involvement of multiple healthcare professionals, lack of belief that collaborative practice will improve patient care, and lack of confidence in the pharmacist’s knowledge or skills to take their advice on patient care issues.

**Table 8:** Barriers to collaborative practice *(in descending order according to percentage agreeing)* (n = 447).

Barrier	Pharmacists(n = 230) n (%)	Physicians (n = 217) n (%)	Total (n = 447) n (%; 95% CI)	P value
Lack of time	188 (81.7)	188 (86.6)	376 (84.1; 80.3–87.3)	0.20
Lack of financial compensation	179 (77.8)	162 (74.7)	341 (76.3; 72.0–80.1)	0.50
Lack of face-to-face communication	177 (77.0)	131 (60.4)	308 (68.9; 64.4–73.1)	<0.001
Involvement of multiple health care providers, resulting in possible fragmentation of care	174 (75.7)	134 (61.8)	308 (68.9; 64.4–73.1)	0.002
Need to deal with multiple healthcare professionals	107 (46.5)	96 (44.2)	203 (45.4; 40.8–50.2)	0.70
Concern regarding liability over shared responsibility	96 (41.7)	76 (35.0)	172 (38.5; 34.0–43.2)	0.17
Concern regarding liability over shared information	89 (38.7)	74 (34.1)	163 (36.5; 32.0–41.1)	0.36
Lack of belief that collaborative practice will improve patient care	49 (21.3)	24 (11.1)	73 (16.3; 13.1–20.2)	0.005
Lack of confidence in the pharmacist’s knowledge or skills to take their advice in patient care issues	42 (18.3)	23 (10.6)	65 (14.5; 11.5–18.2)	0.03

## Discussion

To the best of our knowledge, this is the first study to be conducted in Kuwait to assess the collaborative working relationships between primary care physicians and pharmacists in terms of their attitudes towards and experiences with collaborative practice, preferred methods of communication in collaborative practice, perceptions related to the professional role of pharmacists, areas of potential further collaboration and perceived barriers to collaborative practice. The current results provide a useful baseline quantitative data-set that will assist in the assessment of current physician-pharmacist collaborative practice in primary healthcare, and could be used by the health authorities to design future targeted multifaceted interventions to promote the implementation of optimal physician-pharmacist interprofessional relationships and collaborative practice within clinical practice settings in Kuwait. Also, these findings allow for crucial comparative work with existing and future studies in middle-eastern countries, and worldwide.

The current study provided evidence that although almost all respondents (> 98%) agreed that collaboration specifically between physicians and pharmacists can result in improved patient outcomes, more than half of them had never practised collaboratively in their professional work in the Kuwaiti primary healthcare system. The proportions of physicians and pharmacists in the present study who expressed positive attitudes towards collaborative practice were similar to the findings of a Canadian study [25] but higher than those reported in other studies conducted in Croatia, the USA, Iran, and Slovakia (which ranged between 50% and 71%) [26,27,33,37]. Female respondents expressed significantly higher positive attitudes towards collaborative practice compared to males. This finding is in agreement with other studies, which showed that female generally had more positive attitudes toward interprofessional healthcare teams and interprofessional education than males [41,42]. Potentially, gender might be involved differently to support the development of interprofessional collaboration. This finding could be further explored qualitatively. The high overall positive attitudes of both professions reported in this study would be of importance to provide a basis to establish or enhance interprofessional collaborative practice. The attitudes of healthcare professionals have been one of the significant factors contributing to interprofessional collaboration and a prerequisite for an effective physician-pharmacist collaboration [29]. A study performed in Germany reported that a prerequisite for an effective physician-pharmacist collaboration is positive attitudes on both sides [28].

Despite high overall positive attitudes towards interprofessional collaboration, both professions in the present study had limited experience of working collaboratively, which is consistent with findings from previous studies [7,8,25,26]. These results are distinct from those reported in a study from Slovakia in which the majority of respondents from both professions reported that they collaborate “often” or “sometimes” [27]. This limited collaborative practice could be partly explained by the lack of official policies on interprofessional collaboration and lack of joint undergraduate training courses for both professions. A study conducted among medical and pharmacy students in Kuwait reported that lack of undergraduate education and training in interprofessional collaboration and teamwork is the major perceived barrier to establishing effective physician-pharmacist collaboration [43]. Hence, the present teaching strategies of undergraduate medical and pharmacy students warrant improvement to equip students with knowledge, communication skills, attitudes, and behaviours that will allow them to work collaboratively as future practitioners. Another potential strategy would be the development of interprofessional continuing education programs in case-based learning that target both professions to enhance their partnership in patient care. Offering these opportunities to physicians and pharmacists in the context of continuous professional development activities has been demonstrated to assist in developing their working relationships [44]. Furthermore, the current regulations must be expanded by the health authorities in Kuwait to develop explicit national standards of interprofessional collaborative practice, including the legal frameworks to define and set clear goals and responsibilities for each profession in patient care. Other reasons identified in this study by over two-thirds of respondents as significant barriers to collaborative practice were lack of time, lack of compensation, lack of face-to-face communication, and possible fragmentation of patient care by the involvement of multiple healthcare professionals. These findings highlight the need for interventional strategies to overcome these barriers to allow collaborative practice to become more common in primary healthcare settings in Kuwait. In the present study, physicians were found to be significantly more likely to report that they rarely collaborate with pharmacists and expressed significantly less positive overall perceptions for further collaborations in areas related to the clinical roles of pharmacists. This could be explained by the professional culture of some physicians in which they have traditionally assumed total responsibility for patient outcomes, while being reluctant to involve other healthcare professionals in the clinical decision-making process [21].

Personal forms of communication, face-to-face or via telephone, were indicated by the majority of both professions as the preferred methods of communication. These findings are consistent with those reported in previous similar studies from Canada, Iran and Slovakia [25–27]. Lack of face-to-face communication was also identified by both groups as one of the major barriers to collaborative practice. These results underscore the need to foster personal communication between physicians and pharmacists, since this has been found to have a positive impact on the development of interprofessional relationships [32]. Strategies need to be developed to foster effective communication skills and an environment where both professions would have increased levels of comfortable in-person interactions as often as possible. Face-to-face communication could be achieved by organising joint continuing education programs and conferences. Hashemian et al. suggested the use of cost-effective, time-saving applications that allow video calls to facilitate face-to-face communication to discuss medication-related problems [26]. Pharmacists significantly preferred face-to-face communication compared to physicians. In light of the current model of practice in Kuwait, where physicians have a higher level of medical hierarchy with inherent power over the treatment regimen that they are in control of and have the knowledge and skills needed to make the most possible decisions, which may facilitate their non-adherence to the pharmacists’ recommendations more likely to occur. This necessitates that pharmacists should assume a greater clinical role where it would be more reasonable for them to initiate face-to-face interactions to optimize patient medication therapy.

Pharmacists’ and physicians’ perceptions of the role of pharmacists probably play a vital role in the establishment of collaborative practice in the healthcare system. The present results showed good agreement of both professions on the professional role of pharmacists in terms of helping to manage side effects of drug therapy, helping to improve patient adherence, assisting in dosage adjustment, providing advice regarding drug interactions, and providing drug information to physicians. Physicians perceived dispensing prescriptions as the least important pharmacist’s role, whereas pharmacists believed that it is a major role. Pharmacists viewed that providing advice to physicians regarding modification of a patient’s drug therapy is a minor role, whereas physicians viewed it as one of the most important roles. This discrepancy may be explained partly by the lack of confidence and fear of new responsibilities among some pharmacists, and this may adversely affect their perceptions. Another potential reason is the lack of official policies on clinical pharmacy practice in Kuwait, which was found to result in pharmacists seldom providing clinical services and being perceived solely as drug dispensers [6–8].

The present findings reveal that physicians generally perceived the role to be more based on the cognitive functions of pharmacists and placed less value on the technical role of dispensing medications. These results are distinct from previous studies, which reported that physicians perceive pharmacists’ roles in the more traditional areas of dispensing medications and patient counselling [33,36]. The current findings are consistent with findings of previous studies in which the physicians’ perception of the pharmacists’ roles has changed, with increasing recognition that pharmacists provide incremental value and improve quality in patient clinical care [6,22–24,30]. Furthermore, over four-fifths of both groups agreed that there is the potential for more collaboration in the areas of helping to improve patient adherence, assisting in dosage adjustment, patient counselling, providing drug information to help select a medication, helping in the management of side effects, and making recommendations to modify a patient’s drug therapy.

The present study demonstrated the close role perceptions of both professions and their high level of agreement on the potential for further collaboration in the clinical roles of pharmacists, which would be of importance to the establishment of collaborative practice in the primary healthcare settings of Kuwait. The overall positive perceptions of the potential for more collaboration was found to be significantly higher among those aged < 40 years compared to those aged ≥40 years, which is consistent with previous studies [27,45]. This could be explained by the fact that some young physicians were trained in North America and were exposed to clinical pharmacy practice, which increased their comfort and confidence to collaborate with pharmacists to provide optimal patient care. Other reasons include the recent graduates from Kuwait’s Faculty of Pharmacy, for whom the concept of pharmaceutical care is comprehensively covered into their education and training; and the return of young scholars from abroad with postgraduate degrees in clinical pharmacy [6]. Furthermore, the younger respondents might have a proactive and open approach towards collaborative teamwork [45]. These factors may have contributed to the higher overall positive perceptions of the younger healthcare professionals towards more collaboration.

In order to guarantee successful delivery of interprofessional collaboration, it is essential to identify barriers that would complicate its implementation and design interventions to overcome them. Interestingly, both professions agreed that the most important barriers towards physician-pharmacist collaboration were lack of time, lack of financial compensation, lack of face-to-face communication, and the possible fragmentation of patient care by the involvement of multiple healthcare professionals. These findings are consistent with those of previous studies performed in Canada, Iran, and Slovakia [24–27]. In Canada, both professions indicated lack of time, lack of financial compensation and having to deal with multiple healthcare professionals as the greatest barriers to collaborative practice [24,25]. The lack of face-to-face communication and possible fragmentation of patient care through the involvement of multiple healthcare professionals were identified as the major barriers to collaborative practice in Iran [26]. In Slovakia, lack of compensation, possible fragmentation of patient care through the involvement of multiple healthcare professionals, and concern about time were reported as the greatest barriers to collaborative practice [27].

Strategies should be taken to overcome these potential major barriers, and pharmacists, physicians and health authorities should all take active roles. Pharmacists could have more time if there were better delineation between the roles of pharmacists and pharmacy technicians. If pharmacists were less involved in dispensing and preparation duties, this would “free-up” time for patient care activities [46]. Hence, through re-organization of pharmacy staff duties, a certain amount of time could be routinely scheduled for efficient collaborative practice. The findings of a previous study demonstrated a decrease in the physicians’ workload and an improvement in patient care through a collaborative relationship with clinical pharmacists. The advantage of this collaborative practice was that the clinical pharmacist had the ability to review the medication records, helping to easily identify medication nonadherence, incorrect medication requests, and therapeutic duplications [12]. Therefore, the establishment of collaborative practice in the primary healthcare settings of Kuwait might decrease the physicians’ workload and provide time-effectiveness. Lack of time as a main barrier in this study may be due to the fact that respondents would want remuneration for something they see as an extra activity, since financial compensation is an issue for many pharmacists in international studies. Recent analysis of financial compensation models for pharmacy professional services showed the lack of incentive to provide more or higher-quality service in countries where pharmacists are paid a flat fee to cover all services [47]. It was reported that provision of financial compensation by the government and health insurance companies for collaborative services allowed more collaboration between the healthcare professionals and provided more services to patients [48]. Hence, to support collaborative practice, the health authorities in Kuwait should devise an optimal payment model that reimburses the time needed for physician-pharmacist collaboration.

The possible fragmentation of patient care through the involvement of multiple healthcare professionals could be overcome by the coordination of care through role specification in which each professional has clear shared expectations about their role in patient care. Each practitioner should recognize the complementary role of the other. Role specification has been found to be a vital element in the success of collaborative practices and in offering a framework for interactions [23,31]. Also, it might help in overcoming the time constraints raised by the respondents as a barrier to collaboration. Further qualitative research to elucidate better understanding of barriers and facilitators will help in the adoption and implementation of consistent, evidence-based and integrated physician-pharmacist collaboration in Kuwait.

### Strengths and limitations

The strength of the present study was the adequate sample size and sampling method to produce representative data regarding the study population; hence, its findings can be generalised to healthcare providers in primary healthcare settings in Kuwait. Furthermore, this study fills a gap in the limited existing literature in the Middle Eastern region and provides useful information regarding the collaborative working relationships between the primary care physicians and pharmacists. Limitations of this study include the fact that the study population was selected from primary healthcare settings, as responses from healthcare professionals in secondary and tertiary settings may be different. Hence, the current results may not be representative of all healthcare professionals in Kuwait. Moreover, a further weakness is the use of a Likert scale in the survey, which is prone to central tendency bias (selecting ‘neutral’ answers). Respondents who are honestly uncertain about the topic may select the middle option. Despite the presence of this middle option, more than half of the respondents either agreed or disagreed with most of the survey items, minimising the potential central tendency bias. Another limitation is the social desirability bias: respondents might have offered favourable answers to conform to the more socially accepted view. Also, the cross-sectional nature of the survey represented one point in time and, therefore, does not reflect any changes in respondents’ opinions over time regarding interprofessional collaboration. Furthermore, the relatively low response rate of the physicians (75.9%) compared to pharmacists (99.1%) raises the possibility of non-response bias, and physicians with stronger opinions about interprofessional collaboration might have been more likely to complete the questionnaire. Evidence indicates that physicians’ response rates to questionnaires may be less than ideal, although the reasons for this are unclear [25].

## Conclusions

The present findings provide a better understanding of the professional relationship between primary care physicians and pharmacists through assessing the collaborative working relationships between the two professions regarding their attitudes to and experiences with collaborative practice, preferred methods of communication in collaborative practice, perceptions related to the professional role of pharmacists, areas of potential further collaboration and perceived barriers to collaborative practice. Our findings reveal that despite high overall positive attitudes towards interprofessional collaboration, primary care physicians and pharmacists in Kuwait had limited experience of working collaboratively. The close positive perceptions of the two groups towards pharmacists’ roles and their high level of agreement on the potential for further collaboration in the clinical roles of pharmacists are of importance to the enhancement or establishment of collaborative practice in primary healthcare settings. Both professions agreed that the most important barriers towards physician-pharmacist collaboration were lack of time, lack of compensation, lack of face-to-face communication, and the possible fragmentation of patient care through the involvement of multiple healthcare professionals.

The current results probably have important implications for the enhancement or establishment of physician-pharmacist collaboration in primary healthcare settings in Kuwait. This study highlights the possible interventional strategies for improving this collaborative practice. These include (i) improvement of the undergraduate curricula for medical and pharmacy students to equip them with the knowledge including the expanding clinical roles of pharmacists, communication skills, attitudes, and behaviours required to work collaboratively as future practitioners; (ii) offering joint continuous professional development activities to improve mutual understanding including the expanding clinical roles of pharmacists, communication and trust in their partnership in patient care; (iii) development of explicit national standards of interprofessional collaborative practice, including the legal frameworks to define and set clear goals, role specification and responsibilities for each profession in patient care; (iv) fostering effective communication skills and an environment where both professions would have increased levels of comfortable in-person interactions as often as possible; and (v) provision of an optimal payment model to reimburse the time needed for physician-pharmacist collaboration. Thus, a joint sustained collaboration between the Ministry of Health, the Pharmaceutical and Medical Associations and Kuwait University could promote and implement efficient physician-pharmacist collaborative practice.

## Supporting information

S1 Text (PDF)

S2 Text (PDF)

S1 Raw data (SAV)
