# Supplementary material for: Perspectives of primary care physicians and pharmacists on interprofessional collaboration in Kuwait: A quantitative study
Source: PLoS One. 2020 Jul 20;15(7):e0236114. doi: 10.1371/journal.pone.0236114 (PMC7371165; doi:10.1371/journal.pone.0236114)
Supplement: S2 Text — (PDF) [file pone.0236114.s002.pdf]

Interprofessional Collaborative Practice Survey (Pharmacists)

1. Demographic Characteristics

PLEASE FILL IN OR TICK (√) THE APPROPRIATE ANSWER

1.1 Gender: ☐ Male ☐ Female

1.2 Age (in years): …………………….

1.3 Professional experience (in years): …………….

2. Attitudes and Experience

2.1 Please insert tick (√) in the corresponding box that that BEST fits your level of agreement with each statement

	Strongly agree	Agree	Neutral	Disagree	Strongly disagree
1. I believe collaborative practice between pharmacists and other healthcare professionals improves patient outcomes					
2. I believe collaborative practice between pharmacists and physicians improves patient outcomes					
3. I would consider collaborating with physicians to improve patient outcomes					

2.2 Please insert tick (√) in the corresponding box of the statement that BEST reflects your experience with collaborative practice

1. I have always collaborated with physicians in the past	
2. I have frequently collaborated with physicians in the past	
3. I have sometimes collaborated with physicians in the past	
4. I have rarely collaborated with physicians in the past	
5. I have never collaborated with physicians in the past	

3. Preferred Methods of Communication

Please insert tick (√) in the corresponding box that BEST fits your level of agreement with each method of communication that would work best between you and the physician for a collaborative practice in the primary care setting.

	Strongly agree	Agree	Neutral	Disagree	Strongly disagree
1. Paper					
2. Fax					
3. Telephone					
4. Face-to-Face					
5. Social Media (Twitter, Facebook, Instagram)					

4. Professional role of pharmacists

Please insert tick (√) in the corresponding box that BEST fits your level of agreement with each role of the pharmacist to improve patient care.

	Strongly agree	Agree	Neutral	Disagree	Strongly disagree
1. Providing drug information to physicians to assist in decision-making regarding a specific patient's drug therapy					
2. Dispensing prescriptions					
3. Providing advice to physicians regarding drug interactions					
4. Helping to improve patient adherence					
5. Assisting in medication dosage adjustment					
6. Helping to manage side effects of drug therapy					
7. Patient counselling about their prescriptions					
8. Providing advice to physicians regarding modification of a patient's drug therapy					

5. **Areas for further collaboration between pharmacists and physicians**

Please insert tick (√) in the corresponding box that BEST fits your level of agreement with each area for potential further collaboration with physicians to provide patient care in the primary care setting.

	Strongly agree	Agree	Neutral	Disagree	Strongly disagree
1. Patient counselling					
2. Helping in the management of side effects of drug therapy					
3. Making recommendations to modify a patient's drug therapy patient's drug therapy					
4. Assisting in medication dosage adjustment					
5. Providing drug information to help select a medication					
6. Providing advice regarding drug interactions					
7. Helping to improve patient adherence					

6. **Barriers to collaborative practice between pharmacists and physicians**

Please insert tick (√) in the corresponding box that BEST fits your level of agreement with each statement as a barrier to collaborative practice in the primary care setting

	Strongly agree	Agree	Neutral	Disagree	Strongly disagree
1. Lack of time					
2. Lack of face-to-face communication					
3. Involvement of multiple health care providers resulting in fragmentation of care					
4. Lack of financial compensation					
5. Concern regarding liability over shared patient information					
6. Concern regarding liability over shared patient responsibility					
7. Need to deal with multiple healthcare professionals					
8. Lack of belief that collaborative practice will improve patient care					
9. Lack of confidence in the pharmacists' knowledge or skills to provide advice in patient care issues					
